# The spatial structure of dengue transmission in Kamphaeng Phet Thailand

**DOI:** 10.1371/journal.pntd.0014605

**Published:** 2026-08-31

**Authors:** Sarah H. Ooi, Angkana T. Huang, Surachai Kaewhiran, Aaron Farmer, Darunee Buddhari, Tao Li, Jaykumar Gandhi, Adam R. Pollio, Jun Hang, Matthew A. Conte, Derek A. T. Cummings, Kathryn Anderson, Henrik Salje

**Affiliations:** 1 Department of Genetics, University of Cambridge, Cambridge, United Kingdom; 2 Ministry of Public Health, Nonthaburi, Thailand; 3 Viral Diseases Program, Walter Reed Army Institute of Research, Silver Spring, Maryland, United States of America; 4 Department of Virology, Armed Forces Institute of Medical Sciences (AFRIMS), Bangkok, Thailand; 5 Institute of Cell Biology, Medical University of Innsbruck, Innsbruck, Austria; 6 Department of epidemiology, Johns Hopkins Bloomberg School of Public Health, Baltimore, Maryland, United States of America; 7 Department of Biomedical Engineering, Johns Hopkins Whiting School of Engineering, Johns Hopkins University, Baltimore, Maryland, United States of America; 8 Department of Microbiology and Immunology, SUNY Upstate Medical University, Syracuse, New York, United States of America; University of Florida, UNITED STATES OF AMERICA

## Abstract

**Background:**

Dengue virus (DENV) is a mosquito transmitted arbovirus that represents a severe threat to global populations. The four serotypes of DENV often circulate endemically resulting in many discrete but overlapping transmission chains in space and time, effectively hiding the underlying patterns of transmission, and longer term consequences on local population immunity. Here we combined serotyped case data with sequence data from a single setting over 25 years to characterise the spatial dependence between cases, the number of distinct transmission chains circulating at any time and whether community transmission was followed by serotype-specific local herd immunity.

**Methodology/principal findings:**

In this study, we combined geolocated case data (N = 7,487) from individuals that presented at a provincial hospital in Kamphaeng Phet Province, Thailand alongside a subset of the sequences of the viruses (N = 302) that infected them. We estimated the spatial dependence between case pairs over different temporal and spatial windows, using both serotype and sequence data, and used logistic regression to identify whether host population size or epidemic size were correlated with viral diversity. We found there was spatial dependence between cases at up to 5 km, resulting in localised serotype-specific patterns of immunity in subsequent years. Within a 78.5 km^2^ area there were 9.6 distinct transmission chains at any time and that each 100,000 increase in population size was associated with 9.8 additional chains. While local viral diversity was also correlated with the size of the epidemic within any month, we found diversity only had moderate predictive power to accurately recover epidemic dynamics.

**Conclusions/significance:**

These findings highlight the nuanced and spatially variable ecology of a highly endemic pathogen with substantial immunity. This study also illustrates the strengths of combining multiple data sources to shed light on the nature of DENV transmission.

## Author summary

Dengue virus represents a severe threat to the global population. There are four serotypes of the virus, which often co-circulate in endemic populations. Within any location, there are many overlapping chains of transmission, which makes it difficult to understand the true patterns of transmission and how it links to the spread of the virus. This in turn limits the effectiveness of various interventions to curb the spread of the virus. To fill this knowledge barrier, we apply statistical methods to geocoded and serotyped case data from a 25 year period from the same community, alongside sequenced viruses, to understand the factors that shape transmission and how it changes over time. Through this, we found that the range of transmission is limited to distances of 5km. This transmission leads to the build-up of patchwork immunity to the virus which in turn shapes future transmission. These findings have thus uncovered the transmission patterns hidden by constant circulation of the virus. These results will help guide public health policy with regards to the implementation of spatially-targeted interventions.

## Introduction

Dengue virus (DENV) is an arbovirus that is transmitted by female *Aedes aegypti* and *Aedes albopictus* mosquitoes [1]. Infected individuals may remain asymptomatic or develop febrile symptoms with the severe forms of the disease, dengue hemorrhagic fever and dengue shock syndrome, potentially fatal [2]. There are four serotypes of DENV (DENV1–4) and individuals can get infected by each serotype with infection lasting in long-term homotypic (i.e., the same serotype) immunity but only temporary heterotypic (i.e., different serotype) immunity. The World Health Organisation recorded more than 5 million cases in 2025, with at least 3,000 deaths worldwide [3]. In Thailand, there has been endemic co-circulation of DENV1–4 for decades [4]. The long-term circulation of the virus results in many overlapping transmission chains in space and time, which has largely hidden the underlying patterns of spread within and across communities, limiting our understanding of the drivers of local spread.

It has previously been shown that DENV transmission in Thailand is largely focal, and centered around home locations [5,6]. The movement of *Aedes* mosquitoes is limited [7] and therefore transmission is mainly dependent on human mobility [8]. Further, periods of small-scale transmission could lead to the build up of pockets of localised herd immunity, including in a serotype specific manner, which could drive the future spatial distribution of infection risk [9]. To help understand the underlying spatial structure of pathogen transmission, we can use viral sequence data [10]. Reconstruction of phylogenetic trees can provide insight into the viral diversity of co-circulating viruses, for example through estimating the genetic relatedness between viruses circulating in the same community [6,11]. Knowing the coordinates of case homes provides additional insight into the role that home location may play in pathogen spread. However, only a small proportion of infecting viruses are ever sequenced. Alternatively, in locations where multiple serotypes co-circulate, serotype data, which tends to be more readily available, provides an alternative, albeit cruder, characterisation of local viral diversity and the spatial structure of transmission [6,9]. We can harness this serotype diversity as individuals infected with the different serotypes are not related in transmission.

Alongside providing insight on spatial spread, measures of viral diversity, including Shannon diversity indices, have been suggested to be a predictor of case incidence [12]. This provides an avenue to supplementing traditional case data based surveillance with viral data to understand epidemic dynamics. However, the suitability of this approach to DENV is unknown.

Here we make use of long term case surveillance data and sequences from Kamphaeng Phet, a rural province in northern Thailand, where cases from a 25 year period have been serotyped and geocoded. In addition, the viruses from 302 of these cases have been sequenced providing a unique opportunity to understand the extent to which transmission is focal within rural Thai communities and the extent that diversity data from either serotype or sequences can help predict case dynamics.

## Methods

### Ethical statement

This study was approved by the ethical review boards at the University of Cambridge and the Walter Reed Army Institute of Medical Research.

### Study area

Kamphaeng Phet is a province located in the north of Thailand with a diameter of approximately 105 km. It is predominantly rural with large areas covered with forest. Kamphaeng Phet Province is divided into 78 subdistricts, with subdistricts having a mean area of 110km^2^. The mean distance between centres of subdistricts that share a border is 11.4 km. Data was collected from Kamphaeng Phet Provincial Hospital (KPPH) situated in the capital of the province, Mueang Kamphaeng Phet. It is the largest hospital in the province and receives patients of all ages.

### Case and sequence data

This study utilised the confirmed cases of all four serotypes of dengue from KPPH from 1994 to 2018. The dataset used follows the WHO1997 classification with the cases including laboratory confirmed Dengue Fever (DF), Dengue Hemorrhagic Fever (DHF) and Dengue Shock Syndrome (DSS). For each case, three aspects of information are known: the coordinates of their home sub-district, the day they presented at hospital, and the serotype they were infected with.

We sequenced a subset of isolates obtained from the cases. We randomly selected approximately 10 isolates per serotype-year for sequencing, however in some serotype-years we sequenced more while in others there were insufficient isolates available (S1 Table). The sequencing of the isolates was conducted by the Walter Reed Army Institute of Research using a DENV targeted amplicon sequencing approach as previously described [13]. An Illumina Novaseq 6000 System and the Illumina SP 500-cycle kit, v1.5 were used to perform sequencing. Consensus genome sequences were assembled by mapping to one of four reference sequences (MW946038.1 - DENV1, DQ181803.1 - DENV2, AY676353.1 - DENV3, AY618990.1 - DENV4) using NGS_Mapper [14]. NGS_Mapper variant calling was performed with variant frequencies down to 20%, a minimum Phred score threshold of 25 and a minimum depth of coverage of 10x. Draft consensus genomes were then manually curated IGV [15] and Geneious Prime [16] to ensure removal of any primer induced variants.

### Construction of phylogenetic trees

To investigate the temporal signal of the sequences, we constructed time-resolved phylogenies for each serotype. We used Multiple Alignment using Fast Fourier Transform [17] to obtain a multiple sequence alignment. We then input the multiple sequence alignment into IQ-TREE 3 [18] to construct maximum likelihood trees for each serotype. We implemented both least square dating [19] and ultrafast bootstrapping [20] with 1000 replicates simultaneously during tree construction. We used the general time-reversible substitution model with a gamma parameter for each nucleotide frequency alongside a proportion of invariable sites for all the trees, which was the best model determined by ModelFinder [21]. We input the phylogenetic trees into TempEST [22] to investigate for a temporal signal in the data.

We subsequently used Bayesian Evolutionary Analysis Sampling Trees (BEAST) [23,24] to obtain a separate time-resolved phylogenetic tree for each serotype. BEAST works by averaging over all possible trees, weighting each one by their posterior probability. The uncertainty that is accounted for when using BEAST is important given the limited sequence data. We used the default uninformative priors in BEAST (S2 Table). We used the same substitution model as that for IQ-TREE for all serotypes alongside a random starting tree. We set a strict clock for all the serotypes. All the chains across the serotypes achieved an effective sample size of at least 200 [25]. We used Tracer to assess convergence. The mean rate estimated using BEAST for all serotypes was close to the known rate of substitution for DENV [26] (DENV1: 8.17x10^-4^; DENV2: 6.78x10^-4^; DENV3 genotype 2: 7.79x10^-4^; DENV3 genotype 3: 6.76x10^-4^; DENV4: 8.76x10^-4^).

### Relationship between spatial distance and evolutionary time

We extracted the patristic distance from all sequenced viruses in the time-resolved phylogenetic trees. We then grouped the case pairs by increasing evolutionary time and calculated the mean euclidean distance that separates the home locations of each pair of cases from all pairs within each group. We calculated 95% confidence intervals by repeatedly resampling all pairs with replacement and recalculating the mean distance each time. The 95% confidence intervals were the 2.5 and 97.5 percentiles from the resulting distribution.

### Viral diversity over time

We measured the viral diversity in the sequence data using Shannon Entropy. For each pair of homotypic cases, a Shannon H value was calculated for each base pair in the genome (Equation 1).


$$\begin{array}{c} H=\sum_{A,T,C,G}\left(log(prop)\times prop\right)\times (-\text{1}) \end{array}$$

$$\begin{array}{c} prop=\frac{frequency\;of\;nucleotide}{total\;number\;of\;nucleotides\;} \end{array}$$ (1)

Where (log(prop)×prop)×(−1) is calculated for each of the 4 nucleotides, A, T, C and G. The sum of the 4 values is then taken to be the value of *H*.

log(prop)denotes the natural logarithm of the value of *prop.*

For $prop=\frac{frequency\;of\;nucleotide}{total\;number\;of\;nucleotides\;}$the *total number of nucleotides* is 2 since we are calculating *H* for each pair of sequences. The *frequency of nucleotide* is how often the nucleotide *(A, T, C or G)* appears in the 2 sequences at that position. The value is thus one of 0, 1 or 2.

The average Shannon Entropy (*H*_*w*_) over the length of the genomic window was calculated to account for noise in the data (Equation 2). The genome was split into windows of 25 base pairs (bps), with every window starting every 20 bps. As a result, the genomic windows overlap.


$$\begin{array}{c} H_{w}=\frac{\text{1}}{L}\sum_{k\;in\;window}H_{k} \end{array}$$ (2)

$H_{k}$ is the Shannon entropy between two nucleotides for a single position of the genome and *L* denotes the number of base pairs in the genomic window.

### Probability of cases belonging to the same transmission chain using sequence data

The probability of two cases separated by a distance, *d* of belonging to the same transmission chain is one minus the probability of the two cases separated by distance *d* belonging to different transmission chains. Case pairs are either homotypic (caused by the same serotype) or heterotypic (caused by the different serotypes), thus the probability of a case pair belonging to different chains is the sum of the two scenarios (Equation 3).


$$\begin{array}{c} Pr\left(diff\;chain|d\right)=Pr\left(diff\;chain,\;hom|d\right)+Pr\left(\;diff\;chain,het|d\right) \end{array}$$

$$\begin{array}{c} =\;Pr\left(diff\;chain|hom,d\right)\times Pr\left(hom|d\right)+Pr\left(diff\;chain|het,d\right)\times Pr\left(het|d\right) \end{array}$$

$$\begin{array}{c} =\beta (d)\times \eta (d)+\text{1}\times (\text{1}-\eta (d)) \end{array}$$ (3)

where 𝛽(d) is the probability of a homotypic case pair separated by *d* belonging to a different chain and 𝜂(d) is the probability of case pairs separated by distance *d* being homotypic*.* The probability of a heterotypic case pair belonging to a different chain is 1 since heterotypic case pairs are necessarily unrelated by transmission.

We estimated 𝛽 using the time-resolved phylogenetic trees. We only considered case pairs where both individuals had their infecting virus sequenced. We defined case pairs being sick within a year of each other to be part of different transmission chains if their most recent common ancestor (MRCA) was more than a year before the earlier of the two cases (Equation 4). However, given that not all the cases were sequenced, there is a need to correct for this observation bias. This was done by multiplying 𝛽 by an estimate of true underlying proportion of homotypic case pairs (𝜂). To estimate 𝜂, we only consider case pairs (*case*_*i*_ and *case*_*j*_) where at least one individual has a sequence.


$$\begin{array}{c} \hat{\beta }(d)=\frac{\sum_{i=\text{1}}^{n}\sum_{j=\text{1}}^{n}I\left(d_{ij}\leq d\;km\right)\times I\left(t_{ij}<\;\text{1}\;year\right)\times I\left(G_{ij}>\text{1}\;year\right)\times I(s_{ij}==\text{1})}{\sum_{i=\text{1}}^{n}\sum_{j=\text{1}}^{n}\left(d_{ij}\leq d\;km\right)\times I\left(t_{ij}<\;\text{1}\;year\right)\times I\left(G_{ij}<\infty \right)\times I(s_{ij}==\text{1})} \end{array}$$ (4)

Where $I\left(d_{ij}\leq d\;km\right)$ is an indicator function that is 1 when the distance between *case*_*i*_ and *case*_*j*_ is less than d km and 0 otherwise. $I\left(t_{ij}<\;\text{1}\;year\right)$ is an indicator function that is 1 when the time that *case*_*i*_ and *case*_*j*_ were sick within 1 year of each other and 0 otherwise. $I\left(G_{ij}>\text{1}\;year\right)$ is an indicator function that is 1 when the most recent common ancestor is separated from the earlier of the two cases by more than a year and 0 otherwise. *G*_*ij*_ is the time to the MRCA from the earlier of *case*_*i*_ and *case*_*j*_*.* MRCAs for heterotypic case pairs are taken to be infinite. $I(s_{ij}==\text{1})$ is an indicator function that is 1 when *case*_*i*_ and *case*_*j*_ share the same serotype and 0 otherwise.


$$\begin{array}{c} \hat{\eta }(d)=\frac{\sum_{i=\text{1}}^{N}\sum_{j=\text{1}}^{n}I\left(d_{ij}\leq d\;km\right)\times I\left(t_{ij}<\;\text{1}\;year\right)\times I(s_{ij}==\text{1})}{\sum_{i=\text{1}}^{N}\sum_{j=\text{1}}^{n}\left(d_{ij}\leq d\;km\right)\times I\left(t_{ij}<\;\text{1}\;year\right)} \end{array}$$ (5)

*N* is the number of cases in the whole dataset, while *n* is the number of sequences. $I\left(d_{ij}\leq d\;km\right)$ is an indicator function that is 1 when the distance between *case*_*i*_ and *case*_*j*_ is less than d km and 0 otherwise. $I\left(t_{ij}<\;\text{1}\;year\right)$ is an indicator function that is 1 when the time that *case*_*i*_ and *case*_*j*_ were sick within 1 year of each other and 0 otherwise. $I(s_{ij}==\text{1})$ is an indicator function that is 1 when *case*_*i*_ and *case*_*j*_ share the same serotype and 0 otherwise.

As previously established [6], the number of transmission chains in circulation can be estimated from the reciprocal of the probability of belonging to the same chain.

### Spatial dependence analysis over the short-term

To characterise transmission over short distances, we calculated the proportion of pairs of cases that are sick within 1 month of each other that are homotypic (𝜂(*d*)) estimated using Equation 6.


$$\begin{array}{c} \hat{\eta }(d)=\frac{\sum_{i=\text{1}}^{N}\sum_{j=\text{1}}^{N}I\left(d_{ij}\leq d\;km\right)\times I\left(t_{ij}<\;\text{1}\;month\right)\times I\left(s_{ij}==\text{1}\right)}{\sum_{i=\text{1}}^{N}\sum_{j=\text{1}}^{N}\left(d_{ij}\leq d\;km\right)\times I\left(t_{ij}<\;\text{1}\;month\right)} \end{array}$$ (6)

where *d*_*ij*_ is the distance between the home locations of cases *i* and *j* and *t*_*ij*_ is the time difference in days that cases *i* and *j* were sick. $I\left(d_{ij}\leq d\;km\right)$ is an indicator function that is 1 when the distance between *case*_*i*_ and *case*_*j*_ is less than *d* km and 0 otherwise. $I\left(t_{ij}<\;\text{1}\;month\right)$ is an indicator function that is 1 when the time that *case*_*i*_ and *case*_*j*_ were sick within 1 month of each other and 0 otherwise. $I(s_{ij}==\text{1})$ is an indicator function that is 1 when *case*_*i*_ and *case*_*j*_ share the same serotype and 0 otherwise.

### Probability of cases belonging to the same transmission chain using case data

We used the same method as for the sequence data (Equation 3). We approximated the probability of a homotypic case pair separated by distance *d* belonging to different chains (*P*_*0*_) as the probability of case pairs separated by at least 30 km being homotypic (Equation 7). This assumes cases living far apart from one another belong to different transmission chains. The probability of case pairs being heterotypic is estimated as previously (Equation 6). The reciprocal of probability of belonging to the same chain is the estimated number of transmission chains in circulation at distance *d*.


$$\begin{array}{c} \hat{P_{\text{0}}}=\frac{\sum_{i=\text{1}}^{N}\sum_{j=\text{1}}^{N}I\left(d_{ij}\geq \;\text{3}\text{0}\;km\right)\times I\left(t_{ij}<\;\text{1}\;year\right)\times I(s_{ij}==\text{1})}{\sum_{i=\text{1}}^{N}\sum_{j=\text{1}}^{N}\left(d_{ij}\geq \;\text{3}\text{0}\;km\right)\times I\left(t_{ij}<\;\text{1}\;year\right)} \end{array}$$ (7)

where *N* is the number of cases.$I\left(d_{ij}\geq \;\text{3}\text{0}\;km\right)$ is an indicator function that is 1 when the distance between *case*_*i*_ and *case*_*j*_ is at least 30 km and 0 otherwise. $I\left(t_{ij}<\;\text{1}\;year\right)$ is an indicator function that is 1 when the time that *case*_*i*_ and *case*_*j*_ were sick within 1 year of each other and 0 otherwise. $I(s_{ij}==\text{1})$ is an indicator function that is 1 when *case*_*i*_ and *case*_*j*_ share the same serotype and 0 otherwise.

### Population size around each case

The number of individuals living within a certain radius around a case was estimated using geospatial data from WorldPop [27]. WorldPop uses a Random Forest model to predict the population density. This prediction is subsequently used to redistribute census estimates from a wide range of sources [28] to obtain the population size in each 1 km^2^ grid square in Kamphaeng Phet.

### Number of cases over time

We obtained monthly case counts of dengue hemorrhagic fever (DHF) in KPP from KPPH.

### Population size

We investigated whether the size of the population was associated with the number of transmission chains in circulation via a modified version of Equation 6 (Equation 8).


$$\begin{array}{c} \hat{\eta }\left(d,p_{\text{1}},p_{\text{2}}\right)=\frac{\sum_{i}\sum_{j}I\left(d_{ij}\leq dkm\right)\times I\left(t_{ij}<\text{1}month\right)\times I\left(s_{ij}==\text{1}\right)\times I\left({pop}_{i,d}>p_{\text{1}}\right)\times I\left({pop}_{i,d}<p_{\text{2}}\right)}{\sum_{i}\sum_{j}I\left(d_{ij}\leq dkm\right)\times I\left(t_{ij}<\text{1}month\right)\times I\left({pop}_{i,d}>p_{\text{1}}\right)\times I\left({pop}_{i,d}<p_{\text{2}}\right)} \end{array}$$ (8)

where *pop*_*i,d*_ is the total population within distance *d* of *case i.* $\hat{\eta }\left(d,p_{\text{1}},p_{\text{2}}\right)$ is an estimate of the proportion of case pairs that are homotypic where one individual in the pair has between *p*_*1*_ and *p*_*2*_ people living within distance *d* of them. $I\left(d_{ij}\leq dkm\right)$ is an indicator function that is 1 when the distance between *case*_*i*_ and *case*_*j*_ is within *d* km and 0 otherwise. $I\left(t_{ij}<\text{1}month\right)$ is an indicator function that is 1 when the date that *case*_*i*_ and *case*_*j*_ fell sick are within 1 month of each other and 0 otherwise. $I\left(s_{ij}==\text{1}\right)$ is an indicator function that is 1 when *case*_*i*_ and *case*_*j*_ share the same serotype and 0 otherwise. $I\left({pop}_{i,d}>p_{\text{1}}\right)$ is an indicator function that is 1 when the population size within *d* km of *case*_*i*_ is at least *p*_*1*_ and 0 otherwise. $I\left({pop}_{i,d}<p_{\text{2}}\right)$ is an indicator function that is 1 when the population size within *d* km of *case*_*i*_ is less than *p*_*2*_ and 0 otherwise.

### Number of cases

We analysed whether the number of chains depended on the number of cases (Equation 9) in a similar fashion as with population size (Equation 8).


$$\begin{array}{c} \hat{\eta }\left(d,c_{\text{1}},c_{\text{2}}\right)=\frac{\sum_{i}\sum_{j}I\left(d_{ij}\leq dkm\right)\times I\left(t_{ij}<\text{1}month\right)\times I\left(s_{ij}==\text{1}\right)\times I\left({cases}_{i}>c_{\text{1}}\right)\times I\left({cases}_{i}<c_{\text{2}}\right)}{\sum_{i}\sum_{j}I\left(d_{ij}\leq dkm\right)\times I\left(t_{ij}<\text{1}month\right)\times I\left({cases}_{i}>c_{\text{1}}\right)\times I\left({cases}_{i}<c_{\text{2}}\right)} \end{array}$$ (9)

where *case*_*i*_ is the number of cases recorded in the month that *case*_*i*_ was sick. $\hat{\eta }\left(d,c_{\text{1}},c_{\text{2}}\right)$ is an estimate of the proportion of case pairs that are homotypic where there were between *c*_*1*_ and *c*_*2*_ cases reported in the month that one of the individuals in the pair was sick.$I\left(d_{ij}\leq dkm\right)$is an indicator function that is 1 when the distance between *case*_*i*_ and *case*_*j*_ is within *d* km and 0 otherwise. $I\left(t_{ij}<\text{1}month\right)$ is an indicator function that is 1 when the time that *case*_*i*_ and *case*_*j*_ fell sick are within 1 month of each other and 0 otherwise. $I\left(s_{ij}==\text{1}\right)$ is an indicator function that is 1 when *case*_*i*_ and *case*_*j*_ share the same serotype and 0 otherwise. $I\left({cases}_{i}>c_{\text{1}}\right)$ is an indicator function that is 1 when there were at least *c*_*1*_ cases reported in the month that *case*_*i*_ was sick and 0 otherwise. $I\left({cases}_{i}<c_{\text{2}}\right)$ is an indicator function that is 1 when there were less than *c*_*2*_ cases reported in the month that *case*_*i*_ was sick and 0 otherwise.

### Spatial dependence over the long-term

To investigate transmission over a longer time frame of multiple months or years, we compared the probability of a homotypic case pair being within a certain distance and time window with the probability of observing such a homotypic case pair when the spatial and temporal clustering effects were independent (Equation 10). We also conducted a similar analysis for heterotypic case pairs.


$$\begin{array}{c} \phi_{hom}\left(d_{\text{1}},d_{\text{2}},t_{\text{1}},t_{\text{2}}\right)=\frac{Pr\left(j\epsilon \Omega_{i}(d_{\text{1}},d_{\text{2}},t_{\text{1}},t_{\text{2}})|z_{i}=z_{j}\right)}{Pr\left(j\epsilon \Omega_{i}(d_{\text{1}},d_{\text{2}},\cdot )|z_{i}=z_{j}\right)Pr\left(j\epsilon \Omega_{i}(\cdot ,t_{\text{1}},t_{\text{2}})|z_{i}=z_{j}\right)} \end{array}$$ (10)

Ω_i_(*d*_*1*_,*d*_*2*_,*t*_*1*_,*t*_*2*_) denotes the set of cases within distances *d*_*1*_ and *d*_*2*_ and a time window of *t*_*1*_ and *t*_*2*_. $\phi_{hom}\left(d_{\text{1}},d_{\text{2}},t_{\text{1}},t_{\text{2}}\right)$ is the ratio of the probability of observing a homotypic case pair when space and time effects are not independent in contrast to the probability when space and time effects are independent. $Pr\left(j\epsilon \Omega_{i}(d_{\text{1}},d_{\text{2}},t_{\text{1}},t_{\text{2}})|z_{i}=z_{j}\right)$ is the probability that when *case*_*j*_ shares the same serotype as *case*_*i*_, *case*_*j*_ is within the distance interval of *d*_*1*_ and *d*_*2*_ of *case*_*i*_ and is sick within the time window of *t*_*1*_ and *t*_*2*_ of *case*_*i*_. $Pr\left(j\epsilon \Omega_{i}(d_{\text{1}},d_{\text{2}},\cdot )|z_{i}=z_{j}\right)$ is the probability that when *case*_*j*_ shares the same serotype as *case*_*i*_, *case*_*j*_ lies wi*t*hin *t*he distance interval of *d*_*1*_ and *d*_*2*_ of *case*_*i*_ and is sick at any point in time. $Pr\left(j\epsilon \Omega_{i}(\cdot ,t_{\text{1}},t_{\text{2}})|z_{i}=z_{j}\right)$ is the probability that when *case*_*j*_ shares the same serotype as *case*_*i*_, *case*_*j*_ lies at any distance and is sick within the time window of *t*_*1*_ and *t*_*2*_ of *case*_*i*_. We estimate ϕ_hom_ using Equation 11.


$$\begin{array}{c} {\hat{\phi }}_{hom}\left(d_{\text{1}},d_{\text{2}},t_{\text{1}},t_{\text{2}}\right)=\frac{\left(\sum_{i=\text{1}}^{N}\sum_{j\epsilon \Omega_{i}(\cdot ,\cdot ,\cdot ,\cdot )}z_{ij})(\sum_{i=\text{1}}^{N}\sum_{j\epsilon \Omega_{i}\left(d_{i},d_{j},t_{i},t_{j}\right)}z_{ij}\right)}{\left(\sum_{i=\text{1}}^{N}\sum_{j\epsilon \Omega_{i}\left(d_{i},d_{j},\cdot \right)}z_{ij})(\sum_{i=\text{1}}^{N}\sum_{j\epsilon \Omega_{i}\left(\cdot ,t_{i},t_{j}\right)}z_{ij}\right)} \end{array}$$ (11)

The relative probability for heterotypic case pairs was estimated similarly. $\left(\sum_{i=\text{1}}^{N}\sum_{j\epsilon \Omega_{i}(\cdot ,\cdot ,\cdot ,\cdot )}z_{ij}\right)$ is the number of homotypic (heterotypic) case pairs that are present in the dataset. $\left(\sum_{i=\text{1}}^{N}\sum_{j\epsilon \Omega_{i}\left(d_{i},d_{j},t_{i},t_{j}\right)}z_{ij}\right)$ is the number of homotypic (heterotypic) case pairs that meet two conditions. The first one is that the distance between *case*_*i*_ and *case*_*j*_ is within the interval of *d*_*1*_ to *d*_*2*_. The second is that the time between *case*_*i*_ and *case*_*j*_ falling ill is within the time interval of *t*_*1*_ to *t*_*2*_. $\left(\sum_{i=\text{1}}^{N}\sum_{j\epsilon \Omega_{i}\left(d_{i},d_{j},\cdot \right)}z_{ij}\right)$ is the number of homotypic (heterotypic) case pairs where the dis*t*ance between *case*_*i*_ and *case*_*j*_ is wi*t*hin the interval of *d*_*1*_ to *d*_*2*_. $\left(\sum_{i=\text{1}}^{N}\sum_{j\epsilon \Omega_{i}\left(\cdot ,t_{i},t_{j}\right)}z_{ij}\right)$ is the number of homotypic (heterotypic) case pairs where the time between *case*_*i*_ and *case*_*j*_ falling ill is within the time interval of *t*_*1*_ to *t*_*2*_*.*

### Proportion Heterotypic

To investigate how transmission changes with epidemic size, the number of cases was compared against the proportion of heterotypic case pairs within the same time interval. The proportion of heterotypic case pairs (Equation 12) was taken as the complement of the proportion of homotypic case pairs.


$$\begin{array}{c} \hat{\theta }(t)=\text{1}-\frac{\sum_{i}\sum_{j}I\left(d_{ij}<\text{5}\;km\right)\times I\left(t_{ij}\leq t\;months\right)\times I\left(s_{ij}==\text{1}\right)}{\sum_{i}\sum_{j}I\left(d_{ij}<\text{5}\;km\right)\times I\left(t_{ij}\leq t\;months\right)} \end{array}$$ (12)

$\hat{\theta }(t)$ is the estimated proportion of case pairs that are homotypic. $I\left(d_{ij}<\text{5}\;km\right)$ is an indicator function that is 1 when the distance between *case*_*i*_ and *case*_*j*_ is less than 5km and 0 otherwise. $I\left(t_{ij}\leq t\;months\right)$ is an indicator function that is 1 when the dates that *case*_*i*_ and *case*_*j*_ fell sick are within *t* months of each other and 0 otherwise. $I\left(s_{ij}==\text{1}\right)$ is an indicator function that is 1 when *case*_*i*_ and *case*_*j*_ share the same serotype and 0 otherwise.

To obtain a serotype-specific measure of the proportion of cases that are heterotypic, we considered case pairs where at least one of the individuals has that serotype. To ensure that there was sufficient signal in the data, we conducted the analysis over 5 monthly time intervals. A time window of 5 months was chosen as it is close to the dengue season in the province [29], making this window biologically relevant. To achieve this, the number of cases over each of the 5 months was summed together to obtain a time series of cases. The Pearson correlation was subsequently calculated between the two time series.

### Viral diversity

The relationship between epidemic size and viral diversity was also measured over time windows, *t*. To obtain an estimate of viral diversity per interval, the average Shannon Entropy (*H*_*ij*_) (Equation 2) over windows of the genome was first calculated for each pair of cases in that monthly interval that were within 105 km of each other (the maximum width of the province and thus the maximum possible distance between cases). The window size was 25 bps, starting every 20 bps. The Shannon Entropy between heterotypic case pairs was assumed to be 1 as the sequences between different serotypes are different from each other. The Shannon Entropy is weighted by the underlying proportion of each serotype at time window *t*, which is estimated from the case data. The Pearson correlation was calculated between the time series of cases and the time series of genetic diversity.


$$\begin{array}{c} \overset{―}{H_{t}}=\frac{\sum_{i}\sum_{j}[H_{ij}\times I({ser}_{i}=={ser}_{j})+\text{1}\times I({ser}_{i}\neq {ser}_{j})]\times P({ser}_{i}|t)\times P({ser}_{j}|t)}{N_{t}\times N_{t-\text{1}}} \end{array}$$ (13)

where *ser*_*i*_ and *ser*_*j*_ are the serotypes of *case*_*i*_ and *case*_*j*_ respectively. *N*_*t*_ is the number of sequences in time window *t*. $\begin{array}{c} \overset{―}{H_{t}} \end{array}$ denotes the average shannon entropy across all case pairs at time t. $H_{ij}$ denotes the Shannon Entropy between the sequences for *case*_*i*_ and *case*_*j*_*.*$I({ser}_{i}=={ser}_{j})$ is an indicator function that is 1 when *case*_*i*_ and *case*_*j*_ share the same serotype and 0 otherwise. $I({ser}_{i}\neq {ser}_{j})$ is an indicator function that is 1 when *case*_*i*_ and *case*_*j*_ have different serotypes and 0 otherwise. $P({ser}_{i}|t)$ is the probability of observing serotype of *case*_*i*_ at time *t*. This term was included to correct for the bias that arises when only selecting a subset of cases for sequencing of their infecting virus. $P({ser}_{j}|t)$ is the probability of observing serotype of *case*_*j*_ at time *t*. This term was included to correct for *t*he bias that arises when only selecting a subset of cases for sequencing of their infecting virus.

The size of the time interval used in the analysis was varied between 1 and 6 months in sensitivity analysis.

To calculate diversity by serotype over various time windows, we use a modified version of Equations 1 and 2. We calculate a Shannon H value per base pair in the genome across all sequences of the same serotype in that time window. Subsequently, the average Shannon Entropy (*H*_*w*_) over the genomic window (25 bps) is taken. The sum of the entropy across the windows was taken to be the viral diversity for that time window.

## Results

### Case data overview

**Fig 1:**
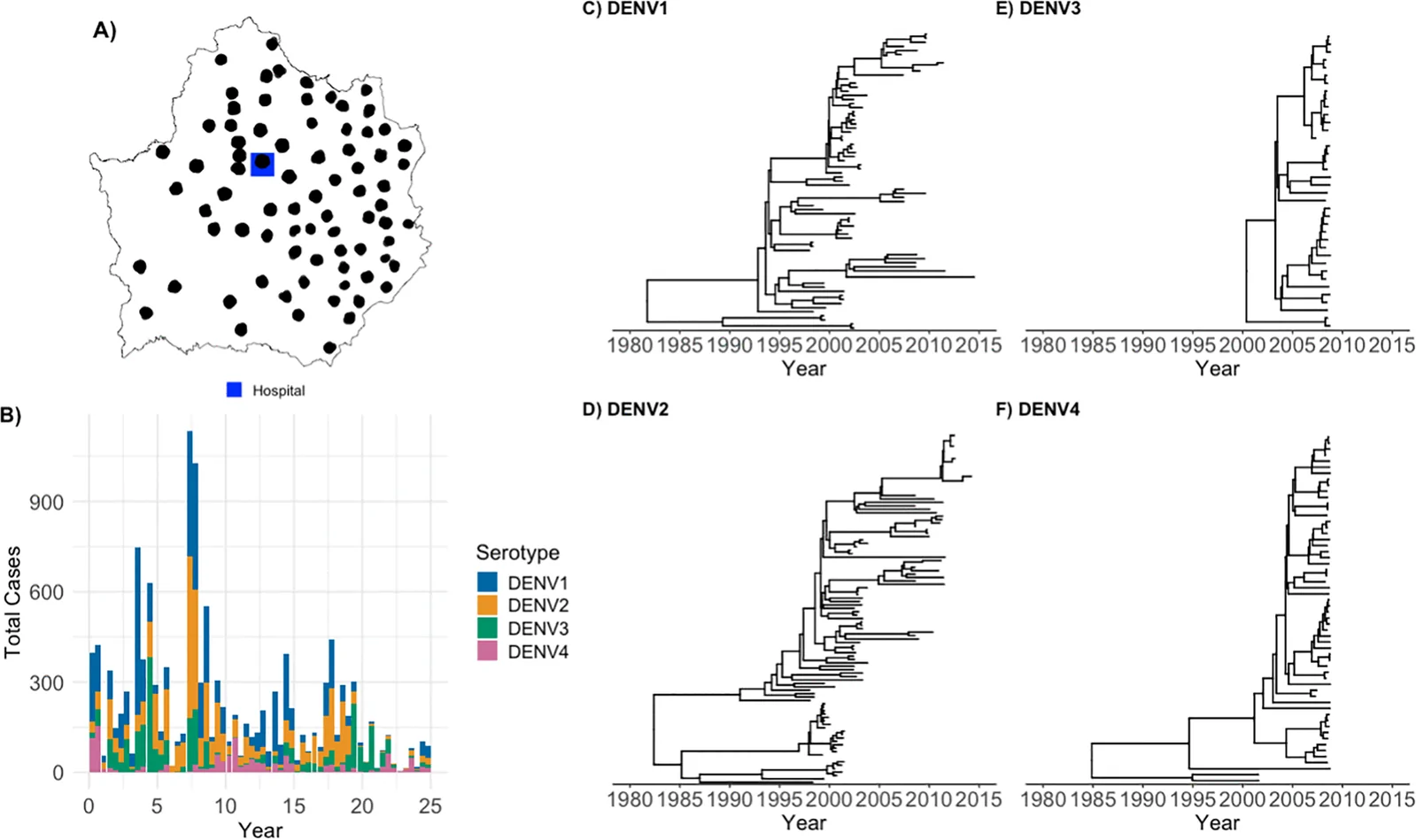
Distribution of case and sequence data. **A)** Map of locations of case data. Map obtained from GADM (https://gadm.org/) using the R package geodata. **B)** Number and proportion of dengue hemorrhagic fever cases by serotype from 1994 to 2018. Each bar is coloured by the proportion of each of the 4 serotypes out of all the cases in a particular 5 month period. The total height of the bars corresponds to the total number of cases in that 5 month period. C-F) Time-resolved phylogenetic trees for each of the 4 serotypes of dengue. The bar at the bottom of each tree shows the time scale of the tree, which is in years. The tips of the trees represent the sequences from the dataset. **C)** DENV1. **D)** DENV2. **E)** DENV3. **F)** DENV4.

We used data from 7,487 PCR-confirmed geocoded DENV cases from between 1994 and 2018 that attended Kamphaeng Phet Provincial hospital as either an outpatient or inpatient (Fig 1A). The mean age of individuals was 15.1 years (range 0–89). Cases came from all four serotypes (29.5% DENV1, 28.5% DENV2, 27.0% DENV3 and 15.0% DENV4) (Fig 1B). Cases were geocoded to their subdistrict, with a mean population of 11,614 per subdistrict in 2006 (which is the middle year out of the 25 years covered in the dataset). A subset of 302 cases had their infecting virus sequenced (24.2% DENV1, 35.1% DENV2, 21.5% DENV3, 19.2% DENV4) spanning from 1998 to 2015 (S1 Table).

### Sequence data identifies spread of virus and number of chains

**Fig 2:**
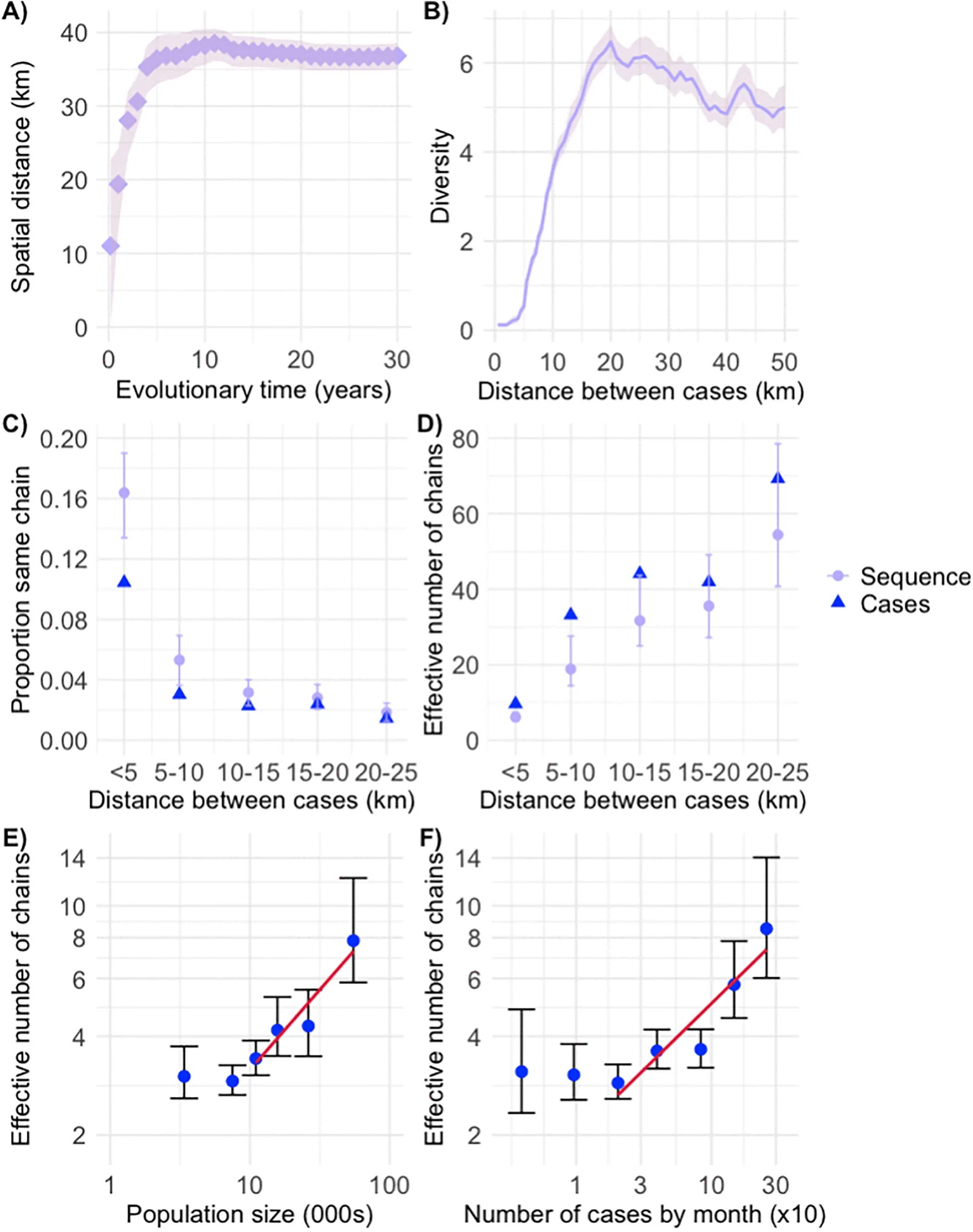
Spatial dependence over time. **A)** Relationship between the evolutionary distance between case pairs and the mean geographic distance between the case pairs. The 95% confidence interval is denoted by the shaded region. **B)** Viral diversity of case pairs within a specific distance. The 95% confidence interval is denoted by the shaded region. **C)** Proportion of case pairs that share the same transmission chain at different distances. The 95% confidence interval is denoted by the error bars. **D)** Estimate of the effective number of transmission chains in circulation. The 95% confidence interval is denoted by the error bars. **E)** Log-scaled effective number of transmission chains at various log-scaled population sizes. The red line denotes the linear line of best fit. **F)** Log-scaled effective number of transmission chains at various log-scaled case counts. The red line denotes the linear line of best fit.

We used time-resolved phylogenies from the sequenced viruses to explore the spread of the virus across the province (Fig 1C-1F). We found pairs of viruses separated by under 1 year in evolutionary time were an average of 19.4 km apart (95%CI: 13.2-24.2), rising to 36.4 km (95%CI: 33.0-39.4) for pairs separated by <5 years, comparable with the mean distance between all pairs of sequenced viruses in our dataset (38.1 km) (Fig 2A). The Shannon Entropy index, an alternative metric of viral diversity, exhibited similar patterns of spatial dependence, with diversity increasing from 0.36 (95%CI: 0.15-0.66) for viruses separated by <5 km to 2.5 (95%CI: 1.68-3.58) for viruses separated by 10 km (Fig 2B).

Consistent with prior work, we considered that pairs of viruses of the same serotype with an MRCA within the same year were part of the same transmission chain [6]. We found that the probability that a pair of cases were part of the same transmission chain fell from 0.16 (95%CI: 0.13-0.19) when separated by less than 5 km to 0.05 (95%CI: 0.03-0.06) when separated by distances between 5 km and 10 km (Fig 2C). The reciprocal of the probability that a pair of individuals are part of the same transmission chain represents an estimate of the effective number of transmission chains circulating within that area (Fig 2D). We found that there were an average of 6.1 distinct chains within 5 km of a case (95%CI: 5.26-7.46), equivalent to an area of 79 km^2^, rising to 18.8 at distances of 10 km (95%CI: 14.4-27.6), equivalent to an area of 314 km^2^.

### Serotype data as an alternative metric of number of chains

We next used the serotyped cases as an alternative metric of the spatial dependence among cases. We found that the probability that case pairs were homotypic if separated by <1 km was 0.66 (95% confidence intervals [CI]:0.64-0.68) (S1A Fig), decreasing to 0.58 (95%CI: 0.56-0.61) at 5 km and to 0.44 for pairs separated by >30 km (95%CI: 0.42-0.45). We assumed that pairs of cases separated by more than 30 km were unlikely to be ever part of the same transmission chain, and therefore represented the underlying probability of unrelated pairs of cases being homotypic. The basis of this assumption was that the proportion of homotypic case pairs remained almost constant beyond this distance (S1A Fig). This assumption allowed us to translate our estimates of the probability into independent estimates of the probability that a pair of cases were part of the same transmission chain for increasing spatial distances (Fig 2C). Using this alternative metric, we found that there were an average of 9.6 distinct chains within 5 km of a case (95%CI: 9.4-9.8), rising to 33.1 at distances of 10 km (95%CI: 31.2-35.3).

The large number of serotyped cases allowed us to explore whether there were spatial or temporal differences in the homotypic clustering of cases. Using a logistic model fitted using population density and the size of the epidemic (measured by the number of cases), we found that each 1,000 increase in the size of the population per km^2^ was associated with 0.40 times the odds of a pair of cases sick within the same month and living within 5 km of each other being homotypic (95%CI: 0.37-0.42). These findings suggest that with additional individuals living within an area, there are increased opportunities for importations of infections and therefore more independent transmission chains circulate. This would decrease the probability that nearby cases share the same infecting serotype. Further, each 100 case increase in the number of cases reported in a month across the entire province was associated with 0.78 times the odds of being homotypic (95%CI: 0.77-0.80) (S1B Fig). By contrast, there was no statistical significant long term trend in the proportion of cases that were homotypic (S1C Fig). By converting the probability that a pair of cases were homotypic into estimates of the effective number of chains, we found that the size of the population living in a 5 km buffer around each case was associated with the effective number of chains in that area (ρ = 0.86) through a linear model (Fig 2E). We also found a strong positive correlation with the epidemic size in the province in that month (ρ = 0.79) using a linear model (Fig 2F). These results concur with what was observed using the sequence data.

### Long term patterns of immunity

**Fig 3:**
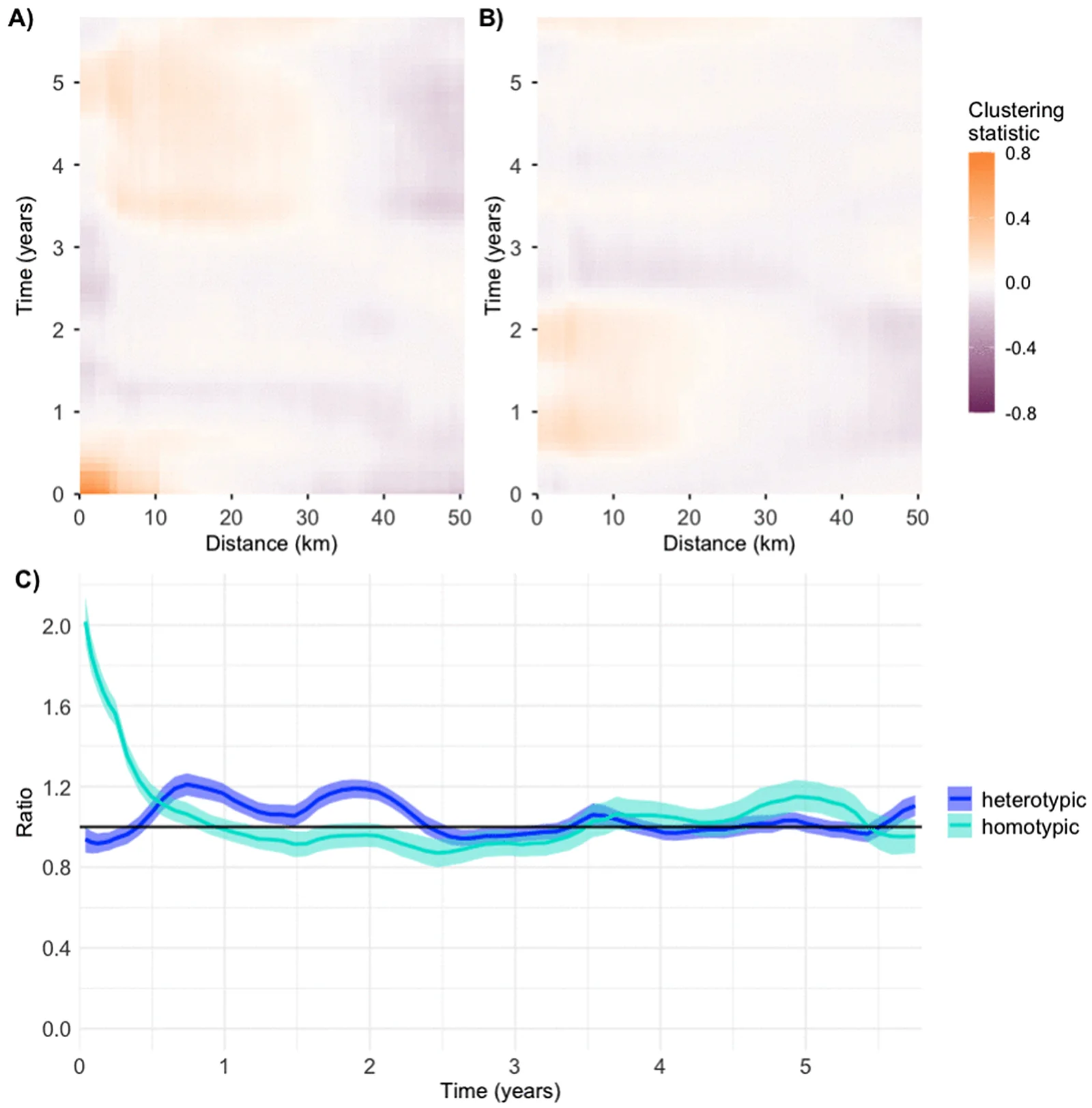
Long-term spatial dependence. **A)** Probability of a homotypic case pair being within a distance and time window relative to the probability observing the homotypic case pair when space and time clustering are independent. The values are shown on a log scale. Values above 0 (orange) would mean that there is more clustering of homotypic case pairs than expected if space and time effects were independent. Values below 0 (purple) would mean that there is less clustering of homotypic case pairs than expected if space and time effects were independent. **B)** Probability of a heterotypic case pair being within a distance and time window relative to the probability when space and time clustering are independent. The values are shown on a log scale. Values above 0 (orange) would mean that there is more clustering of heterotypic case pairs than expected if space and time effects were independent. Values below 0 (purple) would mean that there is less clustering of heterotypic case pairs than expected if space and time effects were independent.C) Probability of a case pair being within 5km and sick within a certain time period of each other relative to the expectation when space and time clustering effects are independent. The black line indicates a ratio of 1. The 95% confidence interval is denoted by the shaded region.

To explore whether our observed pattern of local clustering of cases over short time periods resulted in localised patterns of immunity over long time periods, we compared the probability of observing a homotypic case pair being within a distance and time window to the expected probability of being homotypic if spatial and temporal clustering were independent. We found that homotypic cases had 1.75 times (95%CI: 1.67-1.83) the probability to be located within 5 km and 3 months of each other than if clustering of cases at spatial and temporal scales were independent of each other. This is consistent with short term spatial clustering of homotypic cases (Fig 3A). When we considered a temporal lag of 18–20 months, we found that within 5 km, this statistic fell to 0.93 (95%CI; 0.88-0.99), consistent with a drop in local homotypic cases over this time window. At a longer time lag of 5–6 years, this statistic increased to 1.15 (95%CI: 1.07-1.23) (Fig 3C). We also found that within each serotype, there was a similar rise and decline of clustering of cases over time (S2A-S2D Fig). Heterotypic cases had a different temporal pattern of clustering. Heterotypic cases had 0.92 times (95%CI 0.86-0.97) the probability of being found within 5 km and 3 months of a case, rising to 1.06 (95%CI: 1.01-1.11) when considering a time lag of 18–20 months (Fig 3B). To assess whether there have been secular trends in patterns of localised immunity we repeated the analysis but divided the dataset into two periods (1994–2007 and 2008–2018) (S3 Fig). We found that cases after 2007 exhibited spatial clustering at larger distances than those before 2007, potentially reflecting a role for increasing human mobility resulting in regular transmissions over larger distances.

### Metrics of epidemic size

**Fig 4:**
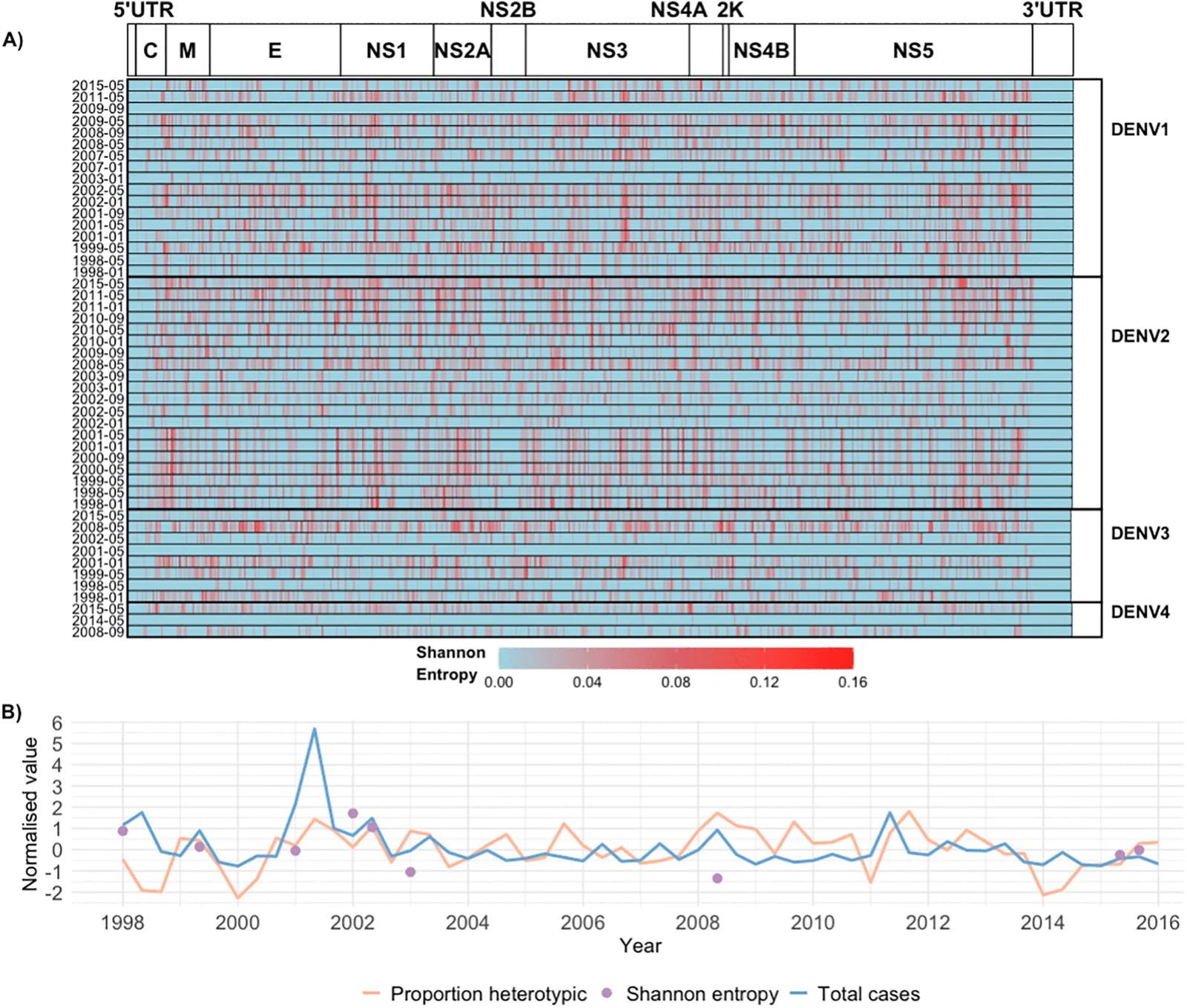
Relationship between transmission and epidemic size. **A)** Viral diversity across the genome over time. The white bar at the top represents the dengue genome that split into regions labelled by the protein that each region encodes. Each coloured tile represents the Shannon Entropy value for a specific genomic window at a specific 5-month window. Low viral diversity is denoted by blue while higher viral diversity values are coloured in red. Each row is the Shannon Entropy across the entire genome for a specific 5-month time window. The bars on the right-hand side of the plot demarcate the diversity values per serotype. The top few coloured rows correspond to the viral diversity across the genome across 5-month time windows for serotype 1. This is followed by the corresponding values for serotypes 2, 3 and 4. The date labels on the left correspond to the starting date of the 5-month window considered. The labels are different per serotype since we only calculated the shannon entropy if there was more than 1 sequence of that serotype in that time window. **B)** Relationship between the number of dengue haemorrhagic fever (DHF) cases, proportion heterotypic case pairs and the viral diversity over 5 month intervals. The purple points are sparse as we only estimate Shannon Entropy when there are at least 10 sequences for that time point.

It has previously been found that sequence diversity for other pathogens is strongly correlated with epidemic size, and therefore can act as an alternative measure of the infection burden in a community [12]. We therefore explored whether dengue sequence diversity was associated with observed dengue epidemic size at any time. We measured viral diversity by calculating the Shannon Entropy over rolling windows of the genome and found high Shannon Entropy across the whole genome for each serotype (Fig 4A). We next compared the average Shannon Entropy from the whole genome for viruses by 5 month windows with the size of an outbreak in the same period, finding a positive correlation (ρ = 0.38) (Fig 4B). As an alternative metric of viral diversity, we compared the proportion of case pairs within 5 km that were heterotypic and found a modest correlation with the number of cases at that time (ρ = 0.24). If we restricted the proportion heterotypic to time windows where sequence data were available, the correlation changed to 0.15. In a sensitivity analysis, we varied the time interval considered for the sequence and case data, finding a similar level of correlation across the time windows considered (S4B Fig).

## Discussion

In this study, we have utilised a dataset that spans a 25 year period from a rural region of Thailand to explore the ecology of DENV over long time scales. The georeferenced cases in this dataset have allowed us to tease apart the overlapping transmission chains to determine the spread of transmission at the neighbourhood scale. We found that transmission is spatially structured across the region and that variability in serotype specific transmission patterns results in localised pockets of homotypic and heterotypic immunity. We found a significant relationship between the size of an area and the number of independent transmission chains circulating in that area. In particular, we estimate about 6.1 distinct chains circulate within 5 km. In contrast, approximately 100 chains were found to circulate within the same distance in Bangkok [9]. This highlights how patterns of transmission depend on the degree of urbanisation. Further, the number of residents residing in an area was independently positively associated with the number of transmission chains. This finding is consistent with what has been found in Bangkok, highlighting the key role of the number of residents living in a location driving the number of external introductions into a community [6]. Beyond population size, we found that monthly epidemic size was also positively associated with the number of transmission chains.

The long timeframe of the dataset allowed us to identify the cyclical nature of local homotypic and heterotypic herd immunity. We found that on a local level, there was short term clustering of cases over three months of the same serotype, consistent with focal transmission patterns found elsewhere [5]. This period was followed by a reduction in homotypic cases in that locality. This is consistent with local herd immunity in that area for 1–3 years. By contrast, we see an increase in heterotypic cases at time lags of 1–2 years. These patterns of homotypic and heterotypic clustering are consistent with what has previously been found in Bangkok [9]. The time periods of increased and reduced homotypic and heterotypic cases will be driven by a combination of the duration of heterotypic cross-protection, spatial structure of transmission determined by human and mosquito mobility, and the introduction and movement of susceptible individuals [8,30,31].

Our findings of strong spatial dependence between cases over short time frames, by both phylogenetic or serotype metrics, point to home location being central to transmission. This does not mean that infection is happening inside houses, and instead may be occurring in other locations near the home. A recent study at a fine spatial scale found little spatial dependence between adult cases occurring within an urbanised community in Viet Nam [11]). Additional work in South America has highlighted the key role of activity space in determining where infections occur, suggesting infection dynamics, and resulting patterns of local immunity may differ across settings [32].

Beyond characterising transmission, we also investigated whether sequence data and case data could be harnessed to predict epidemics, as has previously been done for SARS-CoV-2 [12]. We used the number of cases within a 5-month window as a measure of epidemic size and found that it was correlated with viral diversity in the region, measured either through serotype or sequence diversity. However, the correlation was ultimately only moderate and likely insufficient to improve our understanding of local epidemics where case data exist. It is unclear why these methods work well for SARS-CoV-2 but less well for DENV. With SARS-CoV-2, only part of the genome had high diversity and was used in predicting epidemics, however, we did not find any part of the DENV genomes with distinct levels of increased diversity. We may also have insufficient numbers of sequences for this long study period. Increased sequencing may yet yield an improved ability to use sequences to predict epidemics. Further, more detailed phylodynamic models may also provide independent avenues to successfully predicting epidemic dynamics [33].

Our study highlights the strengths of combining different data sources to understand disease ecology. In particular, the geocoding of cases provides an important opportunity to capture localised differences in DENV behaviour. Our study also highlights the utility of serotyping cases, especially in areas where multiple serotypes cocirculate. Serotype information is available when cases are confirmed through PCR testing, and represent a metric of viral diversity. While serotype information is cruder than whole genome sequences, the large number of serotyped cases relative to sequences allows us to nevertheless estimate key elements, including the number of circulating transmission chains circulating in an area.

This study is subject to some limitations. While we were able to sequence over 300 genomes, which substantially increases the data on viral diversity from this area, this only represents a small proportion of all cases over this long time series. There will also be underlying spatial biases in healthcare seeking behaviours, such that individuals living further away from the hospital may seek healthcare elsewhere. However, our spatial dependence approaches are largely robust to underlying biases in space and time [9]. We were also not able to ultimately explore the mechanism of spread and immunity and our results are based on correlations only. More mechanistic models, including mosquito ecology and human behaviour would allow more mechanistic insights into spread.

## Conclusion

In conclusion, by analysing data from a single setting over a long time period with both serotype and genomic data, we have been able to uncover underlying transmission dynamics of an endemic pathogen, which would not be possible using case data only. We have revealed how the structure of transmission is shaped by local transmission around the home followed by localised population-level homotypic and heterotypic immunity.

## Supporting information

S1 Fig A) Proportion homotypic case pairs that are sick within 1 month of each other over various distances. The 95% confidence interval is denoted by the shaded region. B) Odds ratio for population and epidemic size obtained from a logistic model. C) Proportion of cases sick within 1 month and 5 km of each other that are homotypic over different years in the dataset. The line depicts a linear regression model fit to the data.
(TIF)

S2 Fig A-D) Probability of a homotypic case pair being within a distance and time window relative to the probability observing the homotypic case pair when space and time clustering are independent for each serotype. A) DENV1. B) DENV2. C) DENV3. D) DENV4.
(TIF)

S3 Fig Comparison between the proportion of case pairs within 5km and 1 month of each other that are homotypic over two separate time periods. The 95% confidence interval is denoted by the shaded region.
(TIF)

S4 Fig A) The average viral diversity over time per region of the genome by serotype. The top row is the genome annotation. B) Sensitivity analysis for the time intervals over which the relationship between epidemic size, proportion heterotypic and viral diversity is measured. C) Relationship between the number of dengue haemorrhagic fever (DHF) cases, proportion heterotypic case pairs and the viral diversity in 2015.
(TIF)

S1 Table Number of sequences per serotype per year. Years not shown in the table do not have any sequences.
(XLSX)

S2 Table Prior distributions used to obtain time-resolved phylogenetic trees. (DOC)

## References

[1] Halstead SB. Dengue. Lancet. 370:1644–52. 10.1016/S0140-6736(07)61687-0 17993365

[2] Khan MB, Yang Z-S, Lin C-Y, Hsu M-C, Urbina AN, Assavalapsakul W, et al. Dengue overview: An updated systemic review. J Infect Public Health. 2023;16(10):1625–42. doi: 10.1016/j.jiph.2023.08.001 37595484

[3] Global dengue surveillance. https://worldhealthorg.shinyapps.io/dengue_global/. Accessed 2026 January 27.

[4] Nisalak A, Clapham HE, Kalayanarooj S, Klungthong C, Thaisomboonsuk B, Fernandez S. Forty years of dengue surveillance at a tertiary pediatric hospital in Bangkok, Thailand, 1973-2012. Am J Trop Med Hyg. 2016;94:1342–7. 27022151 10.4269/ajtmh.15-0337 PMC4889755

[5] Mammen MP, Pimgate C, Koenraadt CJM, Rothman AL, Aldstadt J, Nisalak A, et al. Spatial and temporal clustering of dengue virus transmission in Thai villages. PLoS Med. 2008;5(11):e205. doi: 10.1371/journal.pmed.0050205 18986209 PMC2577695

[6] Salje H, Lessler J, Maljkovic Berry I, Melendrez MC, Endy T, Kalayanarooj S, et al. Dengue diversity across spatial and temporal scales: Local structure and the effect of host population size. Science. 2017;355(6331):1302–6. doi: 10.1126/science.aaj9384 28336667 PMC5777672

[7] Stoddard ST, Forshey BM, Morrison AC, Paz-Soldan VA, Vazquez-Prokopec GM, Astete H, et al. House-to-house human movement drives dengue virus transmission. Proc Natl Acad Sci U S A. 2013;110(3):994–9. doi: 10.1073/pnas.1213349110 23277539 PMC3549073

[8] Salje H, Wesolowski A, Brown TS, Kiang MV, Berry IM, Lefrancq N, et al. Reconstructing unseen transmission events to infer dengue dynamics from viral sequences. Nat Commun. 2021;12(1):1810. doi: 10.1038/s41467-021-21888-9 33753725 PMC7985522

[9] Salje H, Lessler J, Endy TP, Curriero FC, Gibbons RV, Nisalak A, et al. Revealing the microscale spatial signature of dengue transmission and immunity in an urban population. Proc Natl Acad Sci U S A. 2012;109(24):9535–8. doi: 10.1073/pnas.1120621109 22645364 PMC3386131

[10] Dudas G, Carvalho LM, Bedford T, Tatem AJ, Baele G, Faria NR, et al. Virus genomes reveal factors that spread and sustained the Ebola epidemic. Nature. 2017;544(7650):309–15. doi: 10.1038/nature22040 28405027 PMC5712493

[11] Masdin L, Ashall J, Kim Huynh M, Biggs J, Phelan J, Shah S, et al. Evaluating dengue transmission dynamics in an endemic urban setting in Viet Nam: An index-household contact cohort study. Lancet Infect Dis. 2026;26(7):714–25. doi: 10.1016/S1473-3099(26)00052-6 41856153

[12] Hill DT, Schulman R, Caldas IV, Dunham C, Zhu Y, Lamson D. Viral genetic variability in wastewater predicts changes in community infection levels. medRxiv. 2025;:2025.10.24.25338735. doi: 10.1101/2025.10.24.25338735

[13] Maljkovic Berry I, Rutvisuttinunt W, Sippy R, Beltran-Ayala E, Figueroa K, Ryan S, et al. The origins of dengue and chikungunya viruses in Ecuador following increased migration from Venezuela and Colombia. BMC Evol Biol. 2020;20(1):31. doi: 10.1186/s12862-020-1596-8 32075576 PMC7031975

[14] ngs_mapper: Genome Mapping Pipeline. Github. https://github.com/VDBWRAIR/ngs_mapper

[15] Robinson JT, Thorvaldsdóttir H, Winckler W, Guttman M, Lander ES, Getz G, et al. Integrative genomics viewer. Nat Biotechnol. 2011;29(1):24–6. doi: 10.1038/nbt.1754 21221095 PMC3346182

[16] Bioinformatics Software for Sequence Data Analysis. https://www.geneious.com/. Accessed 2026 April 24.

[17] Katoh K, Standley DM. MAFFT multiple sequence alignment software version 7: Improvements in performance and usability. Mol Biol Evol. 2013;30(4):772–80. doi: 10.1093/molbev/mst010 23329690 PMC3603318

[18] Wong T, Ly-Trong N, Ren H, Baños H, Roger A, Susko E. IQ-TREE 3: Phylogenomic inference software using complex evolutionary models. EcoEvoRxiv. 2025. doi: 10.32942/x2p62n PMC13191116 42085559

[19] To T-H, Jung M, Lycett S, Gascuel O. Fast dating using least-squares criteria and algorithms. Syst Biol. 2016;65(1):82–97. doi: 10.1093/sysbio/syv068 26424727 PMC4678253

[20] Hoang DT, Chernomor O, von Haeseler A, Minh BQ, Vinh LS. UFBoot2: Improving the ultrafast bootstrap approximation. Mol Biol Evol. 2018;35(2):518–22. doi: 10.1093/molbev/msx281 29077904 PMC5850222

[21] Kalyaanamoorthy S, Minh BQ, Wong TKF, von Haeseler A, Jermiin LS. ModelFinder: Fast model selection for accurate phylogenetic estimates. Nat Methods. 2017;14(6):587–9. doi: 10.1038/nmeth.4285 28481363 PMC5453245

[22] Rambaut A, Lam TT, Max Carvalho L, Pybus OG. Exploring the temporal structure of heterochronous sequences using TempEst (formerly Path-O-Gen). Virus Evol. 2016;2(1):vew007. doi: 10.1093/ve/vew007 27774300 PMC4989882

[23] Baele G, Ji X, Hassler GW, McCrone JT, Shao Y, Zhang Z, et al. BEAST X for Bayesian phylogenetic, phylogeographic and phylodynamic inference. Nat Methods. 2025;22(8):1653–6. doi: 10.1038/s41592-025-02751-x 40624354 PMC12328226

[24] Ayres DL, Cummings MP, Baele G, Darling AE, Lewis PO, Swofford DL, et al. BEAGLE 3: Improved performance, scaling, and usability for a high-performance computing library for statistical phylogenetics. Syst Biol. 2019;68(6):1052–61. doi: 10.1093/sysbio/syz020 31034053 PMC6802572

[25] Lanfear R, Hua X, Warren DL. Estimating the effective sample size of tree topologies from bayesian phylogenetic analyses. Genome Biol Evol. 2016;8(8):2319–32. doi: 10.1093/gbe/evw171 27435794 PMC5010905

[26] Stica CJ, Barrero RA, Murray RZ, Devine GJ, Phillips MJ, Frentiu FD. Global evolutionary history and dynamics of dengue viruses inferred from whole genome sequences. Viruses. 2022;14(4):703. doi: 10.3390/v14040703 35458433 PMC9030598

[27] Worldpop. WorldPop:: Population Density. https://hub.worldpop.org/geodata/summary?id=49097. Accessed 2026 February 9.

[28] Stevens FR, Gaughan AE, Linard C, Tatem AJ. Disaggregating census data for population mapping using random forests with remotely-sensed and ancillary data. PLoS One. 2015;10(2):e0107042. doi: 10.1371/journal.pone.0107042 25689585 PMC4331277

[29] Wichmann O, Yoon I-K, Vong S, Limkittikul K, Gibbons RV, Mammen MP, et al. Dengue in Thailand and Cambodia: An assessment of the degree of underrecognized disease burden based on reported cases. PLoS Negl Trop Dis. 2011;5(3):e996. doi: 10.1371/journal.pntd.0000996 21468308 PMC3066139

[30] Reich NG, Shrestha S, King AA, Rohani P, Lessler J, Kalayanarooj S, et al. Interactions between serotypes of dengue highlight epidemiological impact of cross-immunity. J R Soc Interface. 2013;10(86):20130414. doi: 10.1098/rsif.2013.0414 23825116 PMC3730691

[31] Sabin AB. Research on dengue during World War II. Am J Trop Med Hyg. 1952;1:30–50. 14903434 10.4269/ajtmh.1952.1.30

[32] Perkins TA, Garcia AJ, Paz-Soldán VA, Stoddard ST, Reiner RC, Vazquez-Prokopec G, et al. Theory and data for simulating fine-scale human movement in an urban environment. J R Soc Interface. 2014;11(99):20140642. doi: 10.1098/rsif.2014.0642 25142528 PMC4233749

[33] Rasmussen DA, Boni MF, Koelle K. Reconciling phylodynamics with epidemiology: The case of dengue virus in southern Vietnam. Mol Biol Evol. 2014;31(2):258–71. doi: 10.1093/molbev/mst203 24150038 PMC3907054

