# Supplementary material for: The spatial structure of dengue transmission in Kamphaeng Phet Thailand
Source: PLoS Negl Trop Dis. 2026 Aug 31;20(8):e0014605. doi: 10.1371/journal.pntd.0014605 (PMC13529023; doi:10.1371/journal.pntd.0014605)
Supplement: Supplementary file 1 [file pntd.0014605.s006.doc]

| Parameter | Prior |
| --- | --- |
| General Time Reversible transition rate | Dirichlet (1,1) distribution |
| Base Frequency | Dirichlet (1,1) distribution |
| Gamma shape | Exponential (2) distribution |
| Proportion of invariant sites | Uniform (0,1) distribution |
| Substitution rate | Continuous-Time Markov Chain rate reference prior distribution which is a Gamma distribution with shape 0.5 and rate equal to tree length [[1]](https://paperpile.com/c/RnKusPLG/R6iT) |
| Root height of tree | Coalescent prior under the assumption of constant population size |
| Coalescent population size | Gamma (0.001,1000) distribution |

**Table S2: Prior distributions used to obtain time-resolved phylogenetic trees.**

[1. Tay JH, Kocher A, Duchene S. Assessing the effect of model specification and prior sensitivity on Bayesian tests of temporal signal. PLoS Comput Biol. 2024;20: e1012371.](https://paperpile.com/b/RnKusPLG/R6iT)
